# Patterning technique for gold nanoparticles on substrates using a focused electron beam

**DOI:** 10.3762/bjnano.6.104

**Published:** 2015-04-22

**Authors:** Takahiro Noriki, Shogo Abe, Kotaro Kajikawa, Masayuki Shimojo

**Affiliations:** 1 Department of Materials Science and Engineering, Shibaura Institute of Technology, 3-7-5 Toyosu, Koto-ku, Tokyo, 135-8548, Japan; 2 Interdisciplinary Graduate School of Science and Engineering, Tokyo Institute of Technology, 4259 Nagatsuta, Midori-ku, Yokohama, 226-8502, Japan

**Keywords:** electron beam, gold, nanoparticle array

## Abstract

We propose a novel patterning technique for gold nanoparticles on substrates that combines a chemical reaction with electron beam irradiation. First, gold nanoparticles are placed in a two-dimensional arrangement on the substrate. Then, particular nanoparticles are fixed on the substrate by irradiation with a focused electron beam to produce a desired pattern. Finally, the unfixed nanoparticles are removed. Using this technique, an array of gold nanoparticles, for example, in the form of a line or patterned over an area, are prepared on the substrate. This technique could contribute to the fabrication of plasmonic devices and other applications that require the controlled placement of gold nanoparticles on substrates.

## Introduction

Plasmonic waveguides and circuits utilizing localized surface plasmon resonance (LSPR) are attracting attention for future optical transmission, sensor, and data processing devices. The development of these LSPR-based structures would lead to a reduction in the size of optical circuits and devices [1–2]. Light energy can be propagated through nanometer-sized wires or through rows of particles due to the LSPR effect. Gold and silver nanowires or particles can be used for such waveguides, as these materials interact with visible light.

Plasmon propagation through nanowires has been experimentally investigated. Sanders et al. [3] showed the propagation of plasmons in silver nanowires and the emission of photons at the end of the nanowires. Branching is considered necessary to make integrated photonic/plasmonic circuits. The plasmon propagation on branched silver nanowires was also experimentally demonstrated [4]. However, most of these experiments were performed using nanowires placed on substrates without regard to their position. It is difficult to produce a branched nanowire with a designed shape and to place the nanowire at a desired position at the nanoscale.

It has been theoretically demonstrated that a plasmon can propagate through a chain of spherical metal nanoparticles [5]. According to these results, the nanoparticles should be placed at designated positions and close enough to neighboring particles for transmission at the nanoscale. Gwo et al. [6] fabricated nanoparticles in a line as well as in the form of other two- and three-dimensional structures with gold and silver nanoparticles using a nanomanipulator. This technique is fascinating, but it may be a time-consuming process for production of relatively large circuits.

Nanostructures have also been fabricated using focused ion beam- or focused electron beam-induced deposition [1,7]. However, nanostructures made by these techniques generally have low purity as they contain a large amount of carbon, and thus, the structures need to be coated with gold or silver for use in plasmonic devices. Therefore, a manageable, practical, and not too complicated technique for fabricating nanoparticle arrays of a designed shape is needed.

In this paper, we propose a new patterning technique for gold nanoparticles on substrates. The nanoparticles are first placed in a two-dimensional arrangement on a substrate by chemical methods. Thereafter, the desired nanoparticles are immobilized by focused electron beam irradiation, and finally, the unfixed nanoparticles are removed.

## Results and Discussion

In this technique, the sample was made by implementing the following steps:

Step (i): Au nanoparticles are placed on a substrate.

Step (ii): Selected nanoparticles are immobilized on the substrate by electron beam irradiation.

Step (iii): The unfixed nanoparticles are removed from the substrate.

**Figure 1:**
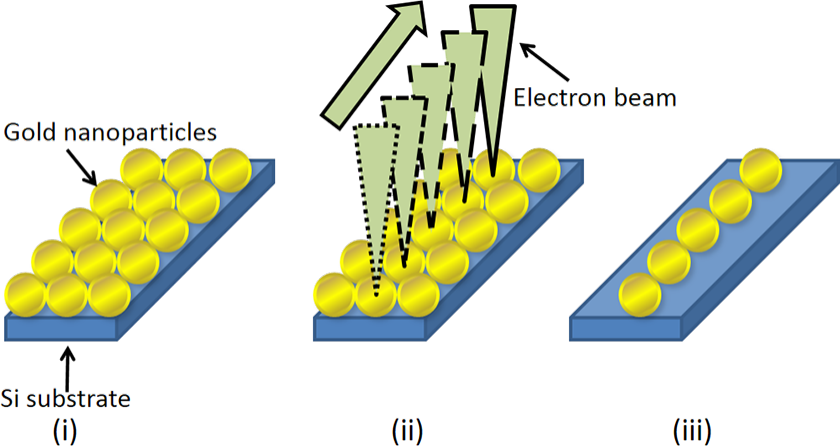
Schematic illustration of the proposed technique. (i) A two-dimensional array of gold nanoparticles is formed on a substrate. (ii) Selected nanoparticles are irradiated with the electron beam to fix these particles on the substrate. (iii) The unfixed particles are washed away, creating an array of nanoparticles.

A schematic illustration of this process is shown in Figure 1. Two methods were employed for step (i), the amine-epoxy method and the amino-undecanethiol method.

**Figure 2:**
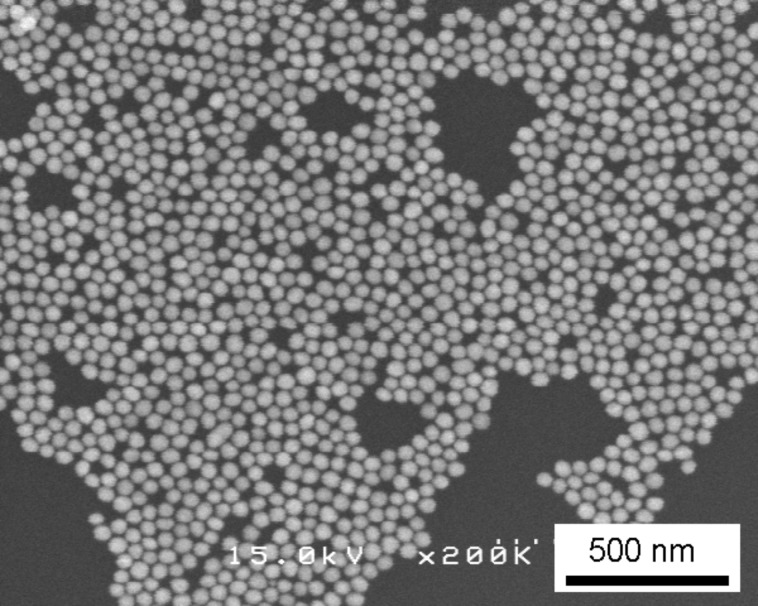
SEM image of the substrate after step (i) using the amine-epoxy method.

Figure 2 shows a scanning electron microscope (SEM) image of gold nanoparticles arranged on the substrate after step (i) using the amine-epoxy method. A similar arrangement technique has been previously published [8]. The length of the molecules attached to the gold particles controlled the distance between neighboring particles. In this method, a two-dimensional close packing of Au particles was partially obtained; however, particles were missing in some areas.

**Figure 3:**
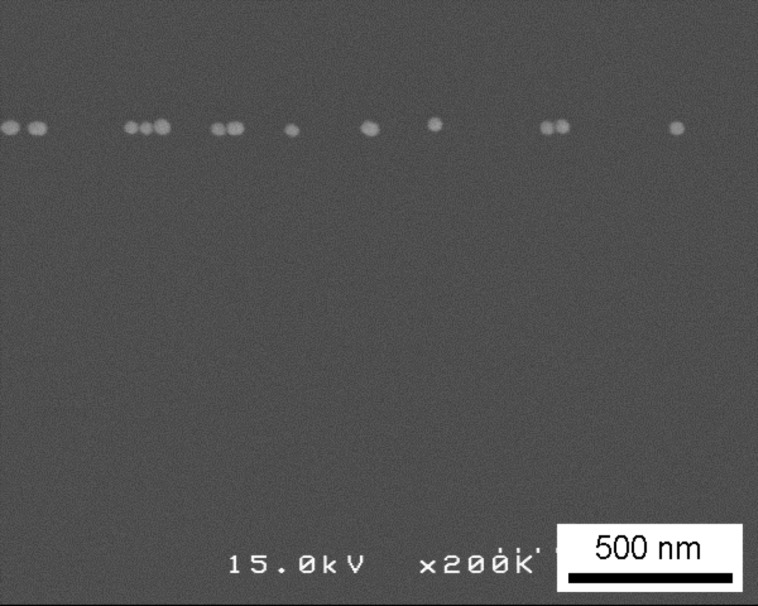
SEM image of the sample demonstrating that a row of nanoparticles was formed after step (iii), wherein the sample was irradiated and washed.

Figure 3 shows a SEM image of the sample after step (iii) where a line of gold nanoparticles remains on the substrate. Since the observation process using SEM immobilizes the nanoparticles in the observation area due to electron beam irradiation, it was necessary to scan the electron beam without prior observation of the substrate. Thus, the area of the sample shown in Figure 3 is different from that of Figure 2. As the position of the focused electron beam can be controlled using a computer, we were able to control and adjust the arrangement of gold nanoparticles, for example, in the form a line.

**Figure 4:**
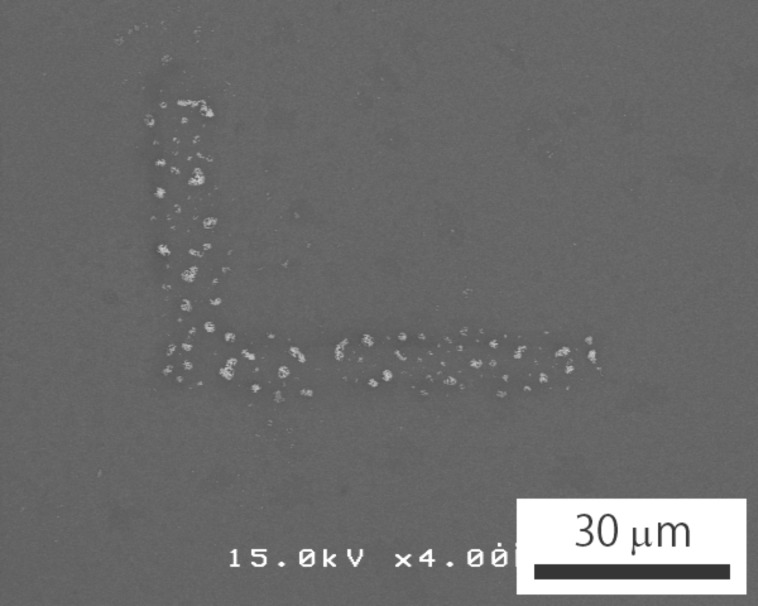
SEM image of the sample irradiated over a relatively large, L-shaped area.

Figure 4 shows a SEM image of the sample that was irradiated over a relatively large, L-shaped area and processed after step (iii). Some close packed regions were left in the irradiated L-shaped area. Although the original distribution was not perfect and many particles were missing in the L-shaped area, it is demonstrated that the electron beam irradiation can immobilize the nanoparticles over a large area on the substrate.

**Figure 5:**
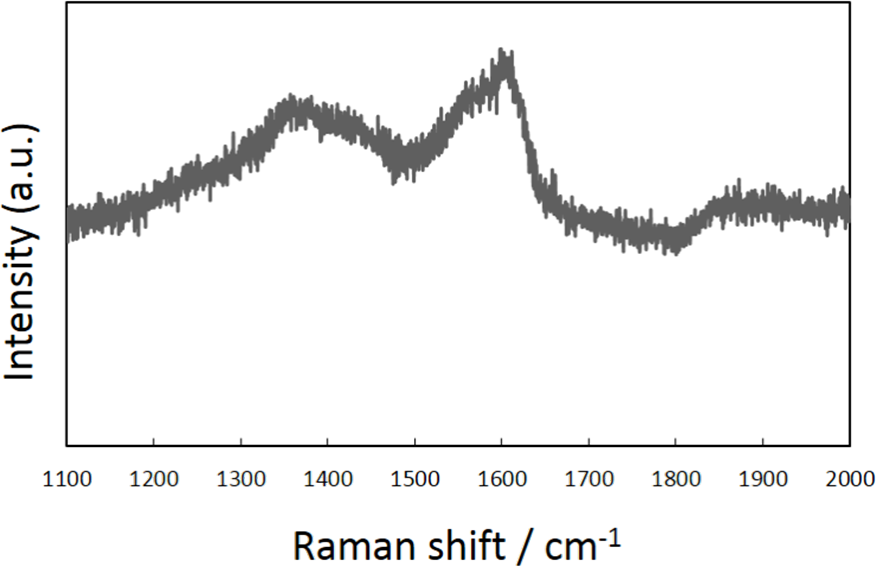
Raman shift measured for the sample, providing evidence of the existence of amorphous carbon.

**Figure 6:**
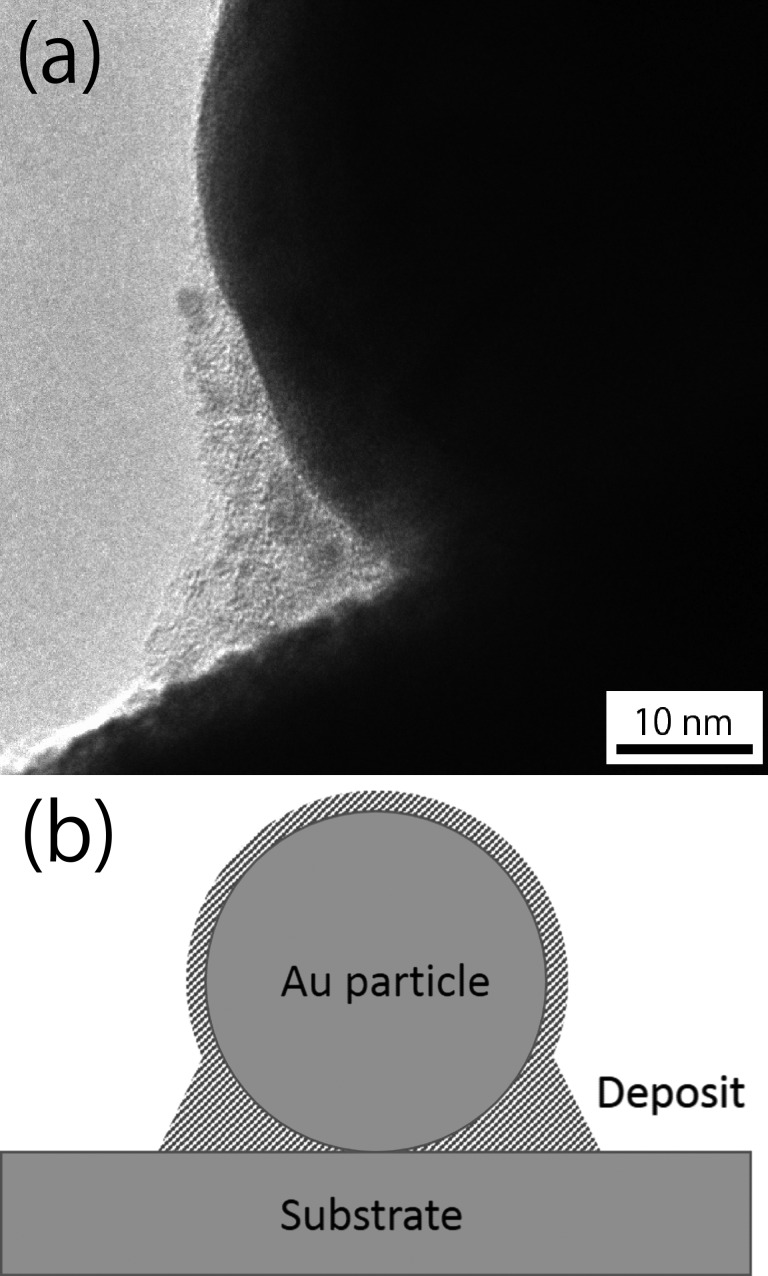
TEM image of the amorphous deposit formed around/between the particle and the substrate (a), and schematic illustration of the immobilization mechanism (b).

To reveal the mechanism of nanoparticle immobilization, Raman spectroscopy was carried out after all process steps of the sample had been performed; however, the electron beam irradiated a large area. Figure 5 shows the Raman shift measured in the area of electron beam irradiation where the G-band (1580 cm^−1^) and D-band (1360 cm^−1^) of carbon are observed. The presence of these bands suggests that amorphous carbon or diamond-like carbon exists on the specimen. It should be noted that no such peaks were observed for the sample without electron beam irradiation (after the steps (i) and (iii)). Figure 6 shows a transmission electron microscope (TEM) image of a nanoparticle on an edge of the widely irradiated specimen, which was tilted in the microscope. An amorphous substance is observed in the gap between the substrate and the particle. A thin amorphous layer is also observed covering the particle. These results suggest that electron beam irradiation decomposes organic molecules formed on the surfaces of the substrate and the nanoparticles, and that the amorphous carbon was formed during step (ii). This amorphous carbon is considered to fix the particles onto the substrate.

The immobilization of the nanoparticle on the substrate surface is considered to occur due to the deposition of amorphous carbon. This amorphous carbon most likely originates from organic molecules around the nanoparticles, as similar mechanisms of decomposition and deposition occur in electron beam-induced deposition (EBID) [9–11]. Fujita et al. reported that amorphous carbon was formed after the irradiation of phenanthrene molecules adsorbed on a substrate by an electron beam [12]. Amorphous carbon was also formed by electron beam-induced deposition using a ferrocene precursor [13]. In our experiment, no precursor gas was introduced into the SEM chamber, however, the nanoparticles were covered with organic molecules and hydrocarbon molecules may have been present in the SEM chamber. Thus, the deposition of amorphous carbon was considered to occur around the particles during the irradiation process, due to the decomposition of organic substances by the high-energy electron beam.

In Figure 3, the spacing between neighboring particles is not uniform. This is probably because the original two-dimensional arrangement was not perfect, as shown in Figure 2. The result of the sample which underwent large area irradiation, as shown in Figure 4, further supports this reason. Thus, another method, the amino-undecanethiol method, was used for step (i).

**Figure 7:**
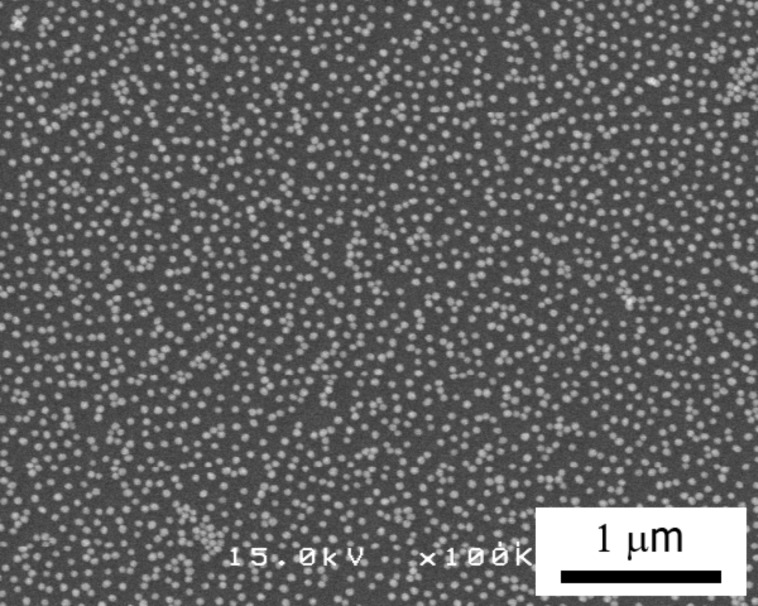
SEM image of the sample after step (i) using the amino-undecanethiol method.

**Figure 8:**
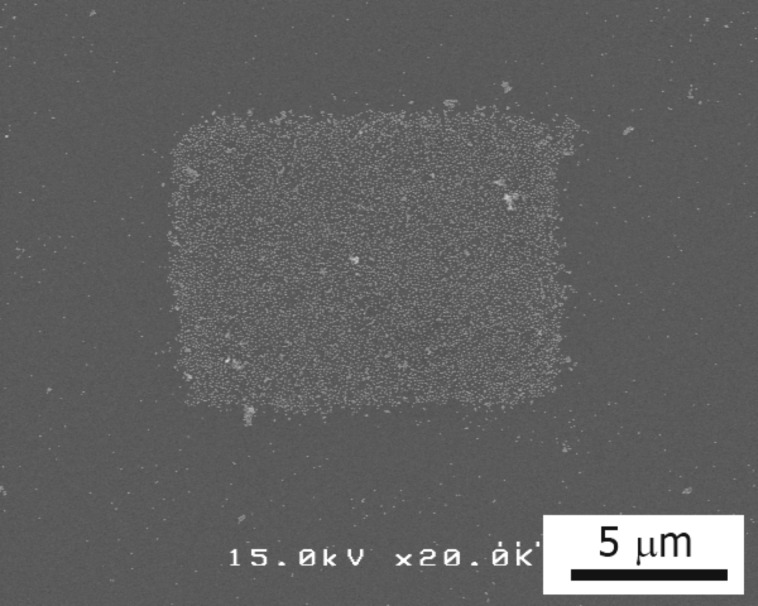
SEM image of the specimen using the amino-undecanethiol method after step (iii), illustrating that the nanoparticles remained in the rectangular area of electron irradiation.

Figure 7 shows a SEM image of the substrate after step (i) using the amino-undecanethiol method. Using this method, the distribution of nanoparticles becomes relatively uniform, as compared with the amine-epoxy method, though the two-dimensional close packing is not obtained. The electron beam was used to irradiate a rectangular area on the substrate prepared using the amino-undecanethiol method. A SEM image of this substrate after step (iii) is shown in Figure 8. Almost all particles remained in the irradiated area on the substrate, while most particles were washed away outside of the irradiated area.

This patterning process for nanoparticles, which combines both chemical and electron beam techniques, could contribute to the fabrication of single electron transistors [14], Fano resonance devices [15] and plasmonic waveguides, as the placement of gold nanoparticles on a substrate could be precisely controlled.

## Conclusion

A novel technique for patterning by controlling the placement of gold nanoparticles on substrates was proposed. The technique combines a chemical reaction with electron beam irradiation. This technique could contribute to the fabrication of nanodevices such as plasmonic waveguides.

## Experimental

### Step (i): amino-epoxy method

A polished Si wafer, with dimensions of about 3 × 1 mm, was used as the substrate. The substrate was immersed in a mixture (1:3 by volume) of hydrogen peroxide (30%) and concentrated sulfuric acid (98%) for 10 h to create a hydrophilic surface [16–17]. The substrate was then immersed in 3-glycidoxypropyltrimethoxysilane for 16 h. During this process, a self-assembled monolayer of molecules with an epoxy group was formed on the Si surface [18].

An ethanol solution (3 mL) of 2-aminoethanethiol (8 mmol/L) was added to a commercially available colloidal gold particle solution (3 mL) and stirred for 1 min. During this process, a monolayer of molecules with an amino group was formed on the gold surface [19].

**Figure 9:**
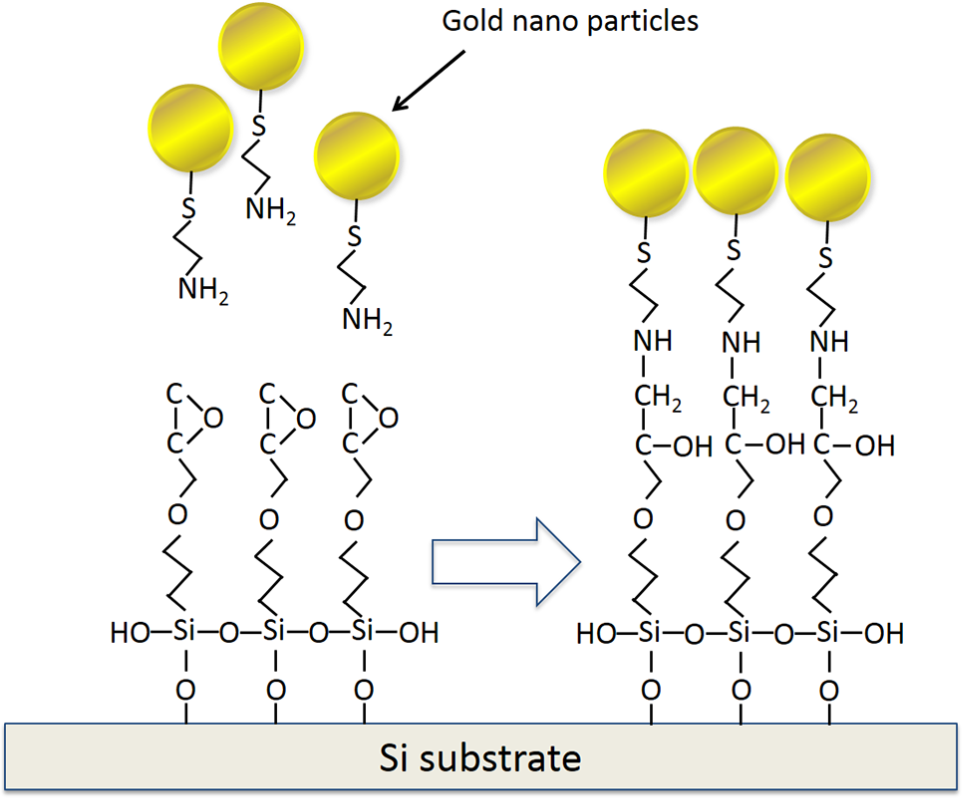
Schematic illustration of the formation of a two-dimensional, dense array of gold nanoparticles on a silicon substrate: step (i).

The substrate was then immersed for 12 h in the colloidal gold solution described above in order to attach the amino group on the gold particles with the epoxy group on the substrate. Afterwards, the substrate was dried in air at room temperature. As a result of these process steps, a two-dimensional array of gold nanoparticles formed on the substrate, as shown schematically in Figure 9.

### Step (i): the amino-undecanethiol method

A thin gold film of 25 nm thickness was formed by sputtering on a Si plate sample. The sample was immersed in amino-1-undecanethiol in an ethanolic solution at a concentration of 1 mM for 3 h. In this process, a self-assembled monolayer was formed [20]. Then, the sample was immersed for 3 h in a colloidal gold solution containing gold particles of 50 nm diameter.

Although this method will not produce a two-dimensional close packing of Au nanoparticles, it will result in the relatively uniform distribution of Au nanoparticles.

### Step (ii): electron beam irradiation

Focused electron beam irradiation on the gold particle-covered substrate was carried out using a SEM equipped with a field emission gun. The accelerating voltage of the electron beam was 15 kV. For a line scan, the irradiation time was about 2 s for each particle. For the L-shaped and rectangular patterns, the electron dose was about 0.07 nC/μm^2^.

This process immobilized the irradiated particles on the substrate by changing the structure of the organic molecules that surrounded the irradiated particles. The detailed mechanism of this step was discussed above.

### Step (iii): removal of unfixed particles

Finally, the irradiated substrate was rinsed in water during ultrasonication with a commercially available surface-active agent for the amine-epoxy method. For the amino-undecanethiol method, an amino-1-undecanethiol in an ethanolic solution was used instead of water. This step removed the unfixed particles on the substrate. Then, the sample, which consisted of the final substrate with immobilized particles, was observed using SEM.

The TEM observation and the Raman spectroscopy measurement were carried out on the specimen to reveal the mechanism of immobilization.
